# Symbiont-mediated mechanisms of insecticide resistance in insects

**DOI:** 10.1093/jisesa/ieag042

**Published:** 2026-05-21

**Authors:** Chao Lv, Qing Xia, Shuhai Yan

**Affiliations:** College of Agricultural and Forestry Science and Technology, Weifang Engineering Vocational College, Weifang, P.R. China; College of Agricultural and Forestry Science and Technology, Weifang Engineering Vocational College, Weifang, P.R. China; College of Agricultural and Forestry Science and Technology, Weifang Engineering Vocational College, Weifang, P.R. China

**Keywords:** insecticide, symbiont, pest management, resistance mechanism, insect

## Abstract

Insecticide resistance threatens sustainable agricultural intensification worldwide. Once regarded solely as a host-encoded evolutionary response, resistance is now known to be strongly influenced by microbial symbionts. Here, we synthesize recent advances showing that vertically and horizontally transmitted bacteria, fungi, and viruses modulate insect–toxicant interactions through 3 intertwined mechanisms: (i) direct enzymatic degradation of the active ingredient inside the gut lumen or hemocoel, (ii) transcriptional or posttranscriptional up-regulation of host detoxification gene networks (cytochrome P450s, carboxylesterases, and glutathione S-transferases), and (iii) epigenetic or immune-mediated priming that accelerates metabolic clearance while dampening insecticide-induced oxidative stress. Symbiont-mediated shifts in toxicity thresholds are context-dependent, varying with host genotype, symbiont strain, insecticide chemistry, and route of exposure. Symbionts may synergize with classical resistance mutations to produce super-resistant phenotypes, or conversely sensitize insects through pro-toxicant bioactivation. We propose an integrative “symbiont–insecticide–host” triangulation framework combining gnotobiotic manipulation, spatial metabolomics, and CRISPR-based microbial genetics to identify causal loci. Microbiome engineering through symbiont replacement, phage therapy or engineered plasmids offers a green resistance-management tool that lowers selection pressure on synthetic chemistries while preserving biocontrol compatibility. Clarifying these microbe-centered pathways is essential for predicting resistance trajectories and for designing durable, next-generation pest-control strategies aligned with food-security and environmental-quality goals.

With social development and population growth, chemical insecticides have been extensively deployed to control agricultural pests and disease vectors in order to meet the rising demand for food and vegetables (Perry et al. 1998, Anaduaka et al. 2023). Owing to their low cost and high efficacy, pesticides quickly became the dominant tactic for pest management; however, prolonged and excessive application has not only selected for robust resistance to a wide array of active ingredients but has also increased pesticide residues in agricultural products (Ffrench-Constant et al. 2004, Bass and Nauen 2023, Liang et al. 2025, Mateos-Fierro et al. 2025). In recent years, detailed investigations of insect–symbiont interactions have successively revealed that microbial symbionts play pivotal roles in nutrient biosynthesis, reproductive regulation, and immune modulation of their insect hosts (Rupawate et al. 2023, Lv et al. 2024, Łukasik and Kolasa 2024, Basit et al. 2025).

## Emergence and Evolution of Resistance

**Fig. 1.**
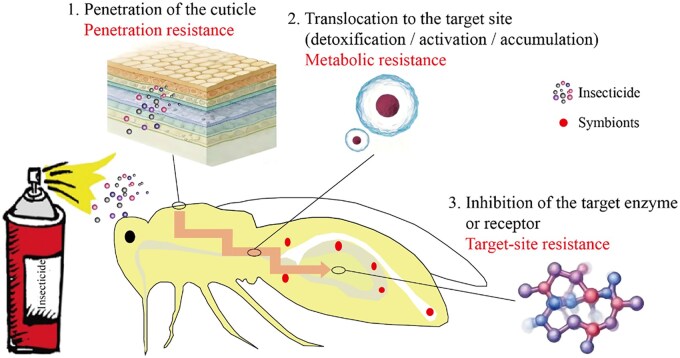
The three core mechanisms of insectcide resistance: reduced penetration, enhanced metabolism, and target-site resistance.

Despite their diverse chemistries, including synthetic organics, botanicals, microbial antibiotics and pheromones, insecticides converge on a conserved intoxication cascade (Fig. 1): cuticular penetration, systemic translocation to the target site where molecules may be detoxified, activated or sequestered and high-affinity disruption of vital enzymes or receptors, ultimately precipitating physiological collapse (Ffrench-Constant et al. 2004, Subhagan et al. 2025). Each node of this cascade represents a potential selection checkpoint for resistance, as detailed below.

The failure of chemical control has ignited a global research surge into resistance mechanisms. Historical records are sobering: by 1984 at least 447 agricultural pest species had evolved resistance to one or more insecticide classes (Georghiou and Mellon 1983), and the number has climbed exponentially, surpassing 500 by 2015 (Sparks and Nauen 2015) and reaching 634 species with 19,520 documented resistance cases by December 2024 (Li et al. 2025b). Resistance is no longer a localized setback but a planetary agro-ecological crisis: yield losses and economic damage now directly undermine food security, while indiscriminate dose escalation to compensate for eroding efficacy escalates pesticide residues, contaminates soils and waterways, and decimates natural enemies and other nontarget organisms (Georghiou and Mellon 1983). Even more ominously, horizontal transfer of resistance genes is forging “super-pest” genotypes that compress the commercial life-cycle of new chemistries from 2 decades to 5 to 7 years, locking agriculture into a self-reinforcing treadmill of high input, intense selection, and enhanced resistance (Ahmad 2007). Confronting this urgent challenge, elucidating the underlying resistance mechanisms and developing green, sustainable counter-strategies have become an imperative for global agriculture.

Resistance is essentially an evolutionary process, in which individuals carrying resistance alleles survive and reproduce under insecticide-mediated selection (Gunning 1991). Notably, populations that evolve resistance to one insecticide class often exhibit cross-resistance to other chemistries sharing the same mode of action, whereas simultaneous tolerance to multiple, structurally unrelated classes gives rise to multiple resistance (Plazzo 1978, Leonard et al. 1988). Such broad-spectrum tolerance reflects the synergistic operation of several resistance mechanisms (Ahmad 2007). Accordingly, and aligned with the intoxication pathway of insecticides, we herein categorize resistance into 3 core mechanisms: reduced penetration (strengthened cuticular barrier), enhanced metabolism (elevated detoxifying enzyme activity), and target-site resistance (mutations that reduce insecticide binding) (Fig. 1).

## Symbiont-Mediated Mitigation of Insecticide Stress: Recent Advances

Vertically transmitted microbial symbionts persist stably across host generations (Kikuchi 2009) and are now recognized as key architects of the nutritional and xenobiotic landscape encountered by insects (Siddiqui et al. 2023, Wang et al. 2023b). First hints of their protective role came from studies on plant-toxin degradation: symbionts routinely detoxify host-ingested secondary metabolites (Ben-Yosef et al. 2015, Cejanavarro et al. 2015), a biochemical capacity logically extendable to synthetic insecticides. The paradigm was formally launched when Boush and Matsumura (1967) demonstrated that bacterial symbionts of the apple maggot could cleave organochlorines, organophosphates, and carbamates, establishing the earliest experimental link between gut microbes and insecticide resistance.

Subsequent work has expanded this framework across taxa and chemistries. *Wolbachia* endosymbionts heighten the sweet-potato whitefly’s survival against acetamiprid and spiromesifen (Ghanim and Kontsedalov 2009); *Enterococcus casseliflavus* colonizing the gut of the pink bollworm and *Escherichia coli* residing in *Manduca sexta* both elevate resistance to *Bacillus thuringiensis* toxins (Broderick et al. 2009); symbionts of the brown planthopper modulate susceptibility to multiple modern insecticides (Li et al. 2025a). Most directly, the gut symbiont *Burkholderia* actively catabolizes the organophosphate fenitrothion, endowing the bean bug *Riptortus pedestris* with field-relevant resistance levels (Kikuchi et al. 2012). Collectively, these findings reposition microbial associates from passive residents to dynamic, evolvable components of the host defence against chemical control.

Yet, symbiont presence does not universally fortify the host. In several systems, the microbiome leaves resistance unchanged or even exacerbates susceptibility. *Wolbachia*, for example, fails to alter *Aedes aegypti* tolerance to bifenthrin, temephos or Bt toxins, whereas *Rickettsia*-infected whiteflies exhibit significantly lower LC_50_ values toward 5 distinct insecticides (Kontsedalov et al. 2008, Pan et al. 2013). Similarly, *Arsenophonus* infection attenuates the resistance phenotype of *Nilaparvata lugens* across multiple chemistries (Pang et al. 2018). Such “sensitizing” effects can arise from metabolic tradeoffs imposed by the symbiont or from its inadvertent conversion of pro-insecticides to more toxic derivatives, as exemplified by *Lactobacillus plantarum* in *Drosophila melanogaster*, which bioactivates chlorpyrifos into its oxon form and thereby overrides host defences (Daisley et al. 2018). Collectively, these contrasting outcomes underscore that the direction (enhancement, attenuation, or neutrality) of symbiont-mediated resistance is contingent upon the idiosyncratic triangle of host genotype, symbiont strain, and insecticide chemistry.

In summary, this section has woven the mode-of-action framework of insecticides into the 3 classical resistance mechanisms: penetration, metabolic detoxification, and target-site modification. It also dissects how symbionts modulate detoxification suites (cytochrome P450s, esterases, and glutathione S-transferases [GSTs]) and immune homeostasis to tip the toxicological balance. Recognizing the contextual nature of these interactions opens a realistic path toward resistance mitigation through precision microbiome engineering, a green alternative to the escalating chemical arms race.

## Symbiont-Orchestrated Defence Against Insecticide Stress: Mechanistic Nodes

A clear mechanistic map linking insecticide modes of action to the corresponding resistance counter-measures is the conceptual bedrock for dissecting symbiont regulatory networks and for designing next-generation control tactics. Resistance evolution is driven by a 3-way interaction: (i) pest biology (generation time, host range, migratory capacity), (ii) insecticide chemistry and application strategy, and (iii) the intensity and consistency of selection pressure. Species with many generations per year and limited dispersal, for example, experience especially strong selection when a single site-specific chemistry is used repeatedly. Insects retaliate with a multilayered defence in which reduced penetration, enhanced metabolism, and target-site insufficiency usually operate in concert. Neonicotinoid resistance in whiteflies and planthoppers exemplifies this synergy, combining *nAChR* mutations with elevated P450 activity (Bass et al. 2011). Below we analyze how microbial symbionts insert themselves into each of these 3 nodes to tilt the toxicological balance.

### Penetration and Behavioral Resistance

Penetration resistance arises from quantitative or qualitative reinforcement of physical barriers, such as cuticle, mid-gut lumen, tracheal walls that slow xenobiotic influx. First formalized in 1962, the phenomenon has since been documented across multiple pest taxa (Forgash et al. 1962, Déglise et al. 2002). A paradigmatic example is the major malaria vector *Anopheles gambiae*: resistant individuals exhibit *CYP4G16*-driven hyperproduction of cuticular hydrocarbons that thicken the epicuticle and reduce the internalization rate of 14C-deltamethrin by approximately 30%, indicating coevolutionary fine-tuning between cuticular barrier reinforcement and pyrethroid resistance (Balabanidou et al. 2016). Behavioral avoidance, including shifts to abaxial feeding, temporary cessation of probing, or nocturnal relocation, also operates at this outermost defensive tier by minimizing insecticide contact. Alone, however, thickened integument or altered behavior rarely confers more than low-level tolerance; high-grade resistance generally requires metabolic or target-site partners. Critically, there is presently no direct molecular evidence that microbial symbionts remodel cuticle architecture. Their hypothetical influence would most likely target chitin-biosynthetic or cuticular-protein modification pathways, a proposition that now demands functional-genomic dissection.

### Metabolic Resistance

Metabolic resistance operates while the toxicant is en route to its target, within the “detoxification–activation–sequestration” phase. Resistant insects typically overexpress or hyperactivate oxidative and hydrolytic enzymes that convert the parent compound into innocuous metabolites; symbionts insert themselves into this pipeline either by donating homologous enzymes or by reprogramming host gene expression.

Field-level selection provides clear ecological evidence. A decade of fenitrothion application enriched soil pseudomonads, bartonellae, and burkholderiae (Tago et al. 2006, Singh 2009) that hydrolyze the P–O-aryl bond, releasing phosphate and low-toxicity phenolics (Ortiz-Hernández et al. 2003). *Burkholderia* isolates from the bean bug, for example, stoichiometrically convert fenitrothion to 3-methyl-4-nitrophenol—an excreted metabolite that the bacterium then exploits as a carbon source (Kikuchi et al. 2012, Sato et al. 2021). Comparable activity occurs in the gut: *Sphingomonas* symbionts of *Aphis gossypii* hydroxylate and nitro-reduce imidacloprid (Lv et al. 2023), whereas the cockroach (*Blatta orientalis*) microbiome mineralizes endosulfan and pyrethroids (Ozdal et al. 2016), and fall armyworm (*Spodoptera frugiperda*) enteric consortia degrade chlorpyrifos, deltamethrin, λ-cyhalothrin, spinosad, chlorantraniliprole, and lufenuron (Almeida et al. 2017, Zhang and Ju 2024). Gut symbionts of *Plutella xylostella* larvae comprising 15 species within *Providencia*, *Pseudomonas*, *Serratia* and 2 additional genera degrade chlorpyrifos to less toxic derivatives (Syamsul Hadi 2021), whereas *Bacillus cereus* and *Achromobacter xylosoxidans* isolated from *Tribolium castaneum* directly catabolize multiple contact grain protectants while simultaneously elevating host detoxification enzyme activities and fitness (Wang et al. 2023a). Conversely, symbionts can reverse the phenotype. Infection with *Arsenophonus* (S-type) downregulates xenobiotic-metabolizing genes in *Nilaparvata lugens*, cutting survival on multiple chemistries (Pang et al. 2018), and *Lactobacillus plantarum* bio-activates chlorpyrifos to its more toxic oxon form, sensitizing *Drosophila melanogaster* (Daisley et al. 2018). Thus, the net contribution of gut microbes to metabolic resistance is bidirectional and contingent on the triad of host genotype, microbial strain, and insecticide structure.

Because oxidation and hydrolysis constitute the 2 principal metabolic routes (Hung et al. 1990), the following sections analyze each pathway separately.

#### Oxidative Detoxification

The cytochrome P450 (CYP) super-family comprising the heme-thiolate mono-oxygenases, cytochrome b5, NADPH-cytochrome P450 reductase, NADH-cytochrome b5 reductase, and ancillary aldehyde oxidases constitutes the primary oxidative engine of insect xenobiotic metabolism (Feyereisen 1999, Nauen et al. 2022). By inserting a single atom of molecular oxygen into lipophilic substrates, CYPs convert insecticides into more polar, readily excretable metabolites and thereby underlie resistance to organochlorines, pyrethroids, and neonicotinoids. Resistance-conferring isoforms cluster within the *CYP4*, *CYP6*, *CYP9*, and *CYP12* clades, whose constitutive overexpression is the biochemical hallmark of many resistant strains (Li et al. 2007). Transcriptional up-regulation of *CYP6G1* alone is sufficient to confer DDT resistance in *Drosophila melanogaster* (Daborn et al. 2002), whereas the contiguous genes *CYP6D1/6D2/6D3* are coordinately overexpressed in pyrethroid- and imidacloprid-resistant houseflies (Mette et al. 2010). The abundance of core symbionts in *Nilaparvata lugens* varies with environmental factors and correlates significantly with the expression of the key detoxification gene *NlCYP6CY3* and with field resistance levels (Zhang et al. 2023). Enrichment of the gut symbiont *Enterococcus mundtii* in *P. xylostella*, which relies on a bacterial P450 to degrade chlorpyrifos, also drives insecticide resistance (Li et al. 2025b). In the sweet-potato whitefly, *CYP6CM1* transcript abundance correlates positively with imidacloprid LC_50_ values across laboratory-selected and field-collected populations (Karunker et al. 2008, 2009); elevated expression of both *CYP6CM1* and *CYP4C64* is similarly associated with field-level neonicotinoid tolerance (Yang et al. 2013). Mechanistically, the MAPK to CREB signaling axis trans-activates the *CYP6CM1* promoter, providing the first direct evidence that a conserved stress-signaling pathway licences P450-mediated resistance (Yang et al. 2020).

Symbionts reinforce oxidative detoxification either by cranking up host *CYP* gene expression or by exporting their own catalytic equivalents. *Wolbachia* illustrates both strategies: in the tea geometric moth *Ectropis grisescens* it transcriptionally boosts 2 P450 genes, doubling bifenthrin tolerance (Gao et al. 2025), while in the 2-spotted spider-mite it upregulates *TuCYP392D2* and the GST *TuGSTd05*, assembling an oxidative-conjugative detoxification pipeline that neutralizes abamectin and related macrocyclic lactones (Ye et al. 2024). Beyond transcriptional lobbying, some microbes perform the chemistry themselves. The gut isolate *E. casseliflavus* EMBL-3 cleaves the amide bridge and dehalogenates chlorantraniliprole, shaving the active concentration reaching host tissues (Zhang and Ju 2024). Likewise, *Citrobacter* sp. CF-BD responds to dichlorvos exposure by overproducing a membrane-bound phosphotriesterase that hydrolyzes the insecticide in the gut lumen (Cheng et al. 2017); concomitant perturbation of host P450 circuitry by the same bacterium further tilts the toxicological balance (Hunter et al. 2017). These examples reveal a versatile symbiont toolkit that either transcriptional priming of host enzymes or direct xenobiotic catabolism, yet the molecular grammar governing the choice between strategies remains largely uncharted.

#### Hydrolytic Detoxification

Carboxylesterases (CarEs), acetylcholinesterase (AChE), GSTs, phosphatases, and amidases constitute the main hydrolytic shield, supplemented by accessory enzymes associated with voltage-gated Na^+^ and Cl^−^ channels (Cruse et al. 2023). At cholinergic synapses, the rapid termination of neurotransmission depends on AChE encoded by ace that hydrolyzes acetylcholine to choline and acetate. Organophosphates (OPs) and carbamates kill by covalently phosphorylating or carbamylating the catalytic serine of AChE, producing irreversible inhibition and persistent excitation (Singh 2009). Proteomic dissection of trichlorfon-resistant larvae uncovered 14 differentially abundant proteins involved in signal transduction (PKA-anchoring protein, DYRK kinase), immunity (hemagglutinin), anabolic/catabolic processes (cathepsin B), oxidative stress (GST, CG7320), energy metabolism (Act57B), and cytoskeletal organization (actin) (Jin et al. 2012). The enrichment of multiple hydrolases and stress-response modules underscores the central role of hydrolytic enzymes in shaping the resistance phenotype.

Insects routinely bolster hydrolytic detoxification by upregulating carboxylesterases (CarEs) and GSTs, and microbial symbionts can amplify the response (Koirala et al. 2022). In *P. xylostella*, an elevated gut CarE titer accelerates indoxacarb hydrolysis (Ramya et al. 2016). *Wolbachia* removal by tetracycline significantly alters superoxide dismutase, peroxidase, carboxylesterase, GST, and mixed-function oxidase activities and reduces resistance in *Laodelphax striatellus* (Liu and Guo 2019) and likewise lowers expression of the GST gene *EscrGST1* and pesticide tolerance in *Eucryptorrhynchus scrobiculatus* (Li et al. 2026). Infection with the facultative symbiont *Hamiltonella defensa* elevates AChE, GST, and CarE activities in the grain aphid above those of uninfected clones, directly expanding hydrolytic capacity (Li et al. 2021). Symbionts do not merely stimulate host enzymes; they contribute their own. *Burkholderia* cells catabolize fenitrothion extracellularly, while gut isolates from the cockroach degrade pyrethroids at rates comparable to host CarEs (Kikuchi et al. 2012, Ozdal et al. 2016, Sato et al. 2021). OP detoxification hinges on a bacterial organophosphorus hydrolase (phosphotriesterase) that cleaves the central P–O–aryl bond, releasing dimethyl phosphate and a nontoxic phenol that the microbe can funnel into central metabolism (Singh and Walker 2006). Thus, host and symbiont hydrolases act as complementary components of a single detoxification network.

Classically, the contribution of this network has been gauged by synergist bioassays (in vivo inhibition of enzyme classes) and by in vitro kinetics comparing resistant and susceptible strains (Hemingway et al. 2002). Integrating population genomics now allows causal variants to be pinpointed, while coupled metatranscriptomic and metabolomic time-series promise to reveal the dynamic cross-talk that governs symbiont–host hydrolytic partnerships. It should be noted that oxidative and hydrolytic detoxification-based resistance are not mutually exclusive; many cases involve both. For example, the gut symbiont *Serratia oryzae* of *Aedes albopictus* not only degrades deltamethrin in vitro but also upregulates host *P450*, *GST*, and *CarE* genes, simultaneously reinforcing oxidative and hydrolytic pathways and thereby enhancing population-level resistance (Wang et al. 2022). A comparable dual mechanism is evident in Venezuelan *A. aegypti* field strains, where elevated mixed-function oxidase and GST activities coincide with multiple *kdr* mutations, indicating that symbiont-primed metabolic resistance can operate alongside classic target-site resistance within the same population (Rubio-Palis et al. 2023).

### Target-Site Resistance

Target-site mutations reduce the sensitivity of the macromolecule to which the insecticide binds, forming the backbone of resistance to organophosphates, carbamates, and pyrethroids (Hemingway 2000). The main players are AChE, the voltage-gated Na^+^ channel, the GABA-gated chloride channel and, more recently, the juvenile-hormone receptor.

#### AChE Mutations

Organophosphates, carbamates, nereistoxin analogs, and nitromethylenes all interrupt cholinergic transmission by blocking AChE (Singh 2009). Insects possess 1 (higher Diptera) or 2 (most other orders) ace genes; point substitutions in either isoform lower enzyme sensitivity. Two mutational patterns predominate: (i) substitutions within the oxyanion hole (Gly to Ser) confer stronger protection against carbamates than organophosphates, whereas (ii) substitutions within the catalytic pocket provide equivalent protection against both classes (Russell et al. 2004). These alleles are now globally distributed in mosquitoes, houseflies, beetles, and aphids and serve as standard molecular diagnostic markers.

#### Na^+^-Channel Mutations

Pyrethroids and DDT bind to the inner pore of the voltage-gated Na^+^ channel, locking it in an open state. A leucine-to-phenylalanine substitution at position 1014 in domain II-S6 (the ­classic *kdr* mutation) sterically hinders insecticide binding and delays channel inactivation, producing nerve-indepo-larization-based resistance (Martinez-Torres et al. 1998, Martinez-Torres et al. 1999). *kdr* and its super-*kdr* companions have been detected in mosquitoes, flies, moths, beetles, and mites worldwide and remain the canonical markers for pyrethroid resistance monitoring (Lee et al. 2000, Brengues et al. 2003).

#### Other Targets

Cyclodienes and fipronil target the GABA-gated chloride channel, while juvenile-hormone analogs require an intact JH receptor; point mutations or downregulation of these targets generate classic target-site resistance. Exactly the same logic applies to the diamide insecticides that lock the ryanodine receptor (RyR) in an open conformation: *Spodoptera frugiperda* evolves resistance primarily through trans-membrane or pore-domain substitutions (I4734M, Y4736S, etc.) that lower ligand affinity without any measurable increase in detoxifying enzymes (Du and Fu 2023). Although the structural consequences of RyR, GABA, and JH-receptor variants are now well documented, virtually nothing is known about whether microbial symbionts can posttranslationally modify these proteins or modulate their expression to influence resistance. Closing this knowledge gap will be critical for designing next-generation chemistries that retain efficacy even in pests whose microbiome provides an additional shield.

## Conclusions and Prospects

Insecticide-resistance research is shifting from a single gene—single chemical paradigm to an ecological network—systems intervention framework. Our synthesis shows that microbial symbionts act as metabolic amplifiers via 2 nonexclusive routes: (i) direct catabolism of the active ingredient by symbiont-encoded enzymes and (ii) transcriptional or posttranscriptional upregulation of host detoxification genes. Their potential to modulate penetration and target-site resistance is emerging, but causal evidence and molecular detail remain almost blank. The field is currently trapped in association rich, mechanism poor territory. Most studies stop at correlative microbiota phenotype links; gnotobiotic rearing and symbiont-gene knock-outs that could establish causality are still scarce. Host genotype, geographic population structure, and environmental stress often flip the outcome of the same symbiont from protection to sensitization or neutrality, making laboratory results hard to extrapolate to the field. Moreover, although meta-transcriptomic and meta-metabolomic inventories are expanding, they lack spatiotemporal resolution; we still cannot quantify the real-time matching of microbe, enzyme, and insecticide within the gut lumen, nerve ganglion, or cuticle.

To escape this association trap, we propose a symbiont insecticide host three-dimensional (3D) pipeline: first, generate dose response surfaces for symbiont positive versus symbiont negative lines across chemistries such as pyrethroids, organophosphates, and neonicotinoids to pinpoint combinations where the microbe significantly raises or lowers tolerance. Second, test multiple host genotypes within each mode-of-action class to determine the generality of the symbiont effect and overlay expression profiles of major resistance loci (kdr, ace-1, CYP6G1, etc.) to build a symbiont–host gene interaction network. Third, integrate degradation pathways, target-site modifications, and immune readouts to extract universal rules and to score the field potential of the symbiont as either a live synergist or a precision sensitizing target. Besides the 3 canonical routes, emerging evidence indicates that sequestration resistance, in which insecticides are bound or vacuole-stored by host or symbiont proteins, should also be overlaid on the same 3D pipeline to test whether microbes also govern the hidden sink that lowers effective toxicant load (Pu and Chung 2024).

Three nested questions must then be answered in succession: is the symbiont effect contingent on host and bacterial genotype; among populations, is resistance divergence driven primarily by the symbiont’s enzymatic repertoire, by copy-number variation in host detox genes, or by their interaction; and does symbiont-conferred resistance intersect or synergize with resistance to biocontrol agents such as *Beauveria bassiana* or entomopathogenic nematodes? Clarifying the universality and manipulability of pest–symbiont associations will allow us to convert “accelerated degradation” into “targeted degradation,” thereby reducing environmental pesticide loads, and to exploit insect–microbe systems for bioremediation and for discovering next-generation active ingredients, ensuring the long-term sustainability of pest management.
